# 168. Testing, Diagnosis, and Incidence of Sexually Transmitted Infections Among People with Substance Use Disorders in the Veterans Health Administration, 2019

**DOI:** 10.1093/ofid/ofab466.168

**Published:** 2021-12-04

**Authors:** Holly Villamagna, Lauren Beste, Joleen Borgerding, Elliott Lowy, Ronald Hauser, Marissa Maier

**Affiliations:** 1 Oregon Health and Science University, Portland, Oregon; 2 VA Puget Sound Health Care System, Seattle, Washington; 3 VA Puget Sound HCS, Seattle, Washington; 4 Yale University School of Medicine, West Haven, Connecticut; 5 VA Portland Health Care System/Oregon Health and Sciences University, Portland, OR

## Abstract

**Background:**

People with substance use disorders (SUDs) are at increased risk of acquiring sexually transmitted infections (STIs.) In response to the syndemic of STIs and SUDs, the Department of Health and Human Services’ 2020 STI National Strategic Plan called for increased STI testing among people with SUDs and integration of testing and treatment into non-traditional settings. Existing data describing STI testing and incidence rates among people with SUDs are limited to single or regional medical centers. National samples are needed to target interventions. We report on STI testing, test positivity, and incidence rates among people with SUDs who receive medical care in the Veterans Health Administration (VHA).

**Methods:**

We performed a retrospective cohort study of individuals with SUDs who received VHA care in 2018 or 2019. Data were obtained from the Corporate Data Warehouse, a national database that includes data from VHA’s electronic medical record. For individuals with alcohol, opioid, cocaine, and/or other stimulant (e.g. methamphetamine) use disorders, we collected demographic data, testing and results for gonorrhea (GC), chlamydia (CT), syphilis, and HIV during 2019. We calculated rates of testing, test positivity, and incidence rates.

**Results:**

Incidence of all four STIs was highest in the other stimulant use disorder group; incidence of syphilis was particularly elevated at 922.4 cases/100K. Veterans with multiple SUDs were three times more likely to be houseless in 2019 than those with a single SUD and had higher incidence of all STIs than those with single SUDs, except for people with other stimulant use disorders. People with alcohol use disorder (AUD) had a higher incidence of GC, CT, and syphilis than those with opioid use disorder despite similar testing rates. Percent positivity for HIV ranged from 0.27% for AUD to 2.0% for other stimulant use disorders.

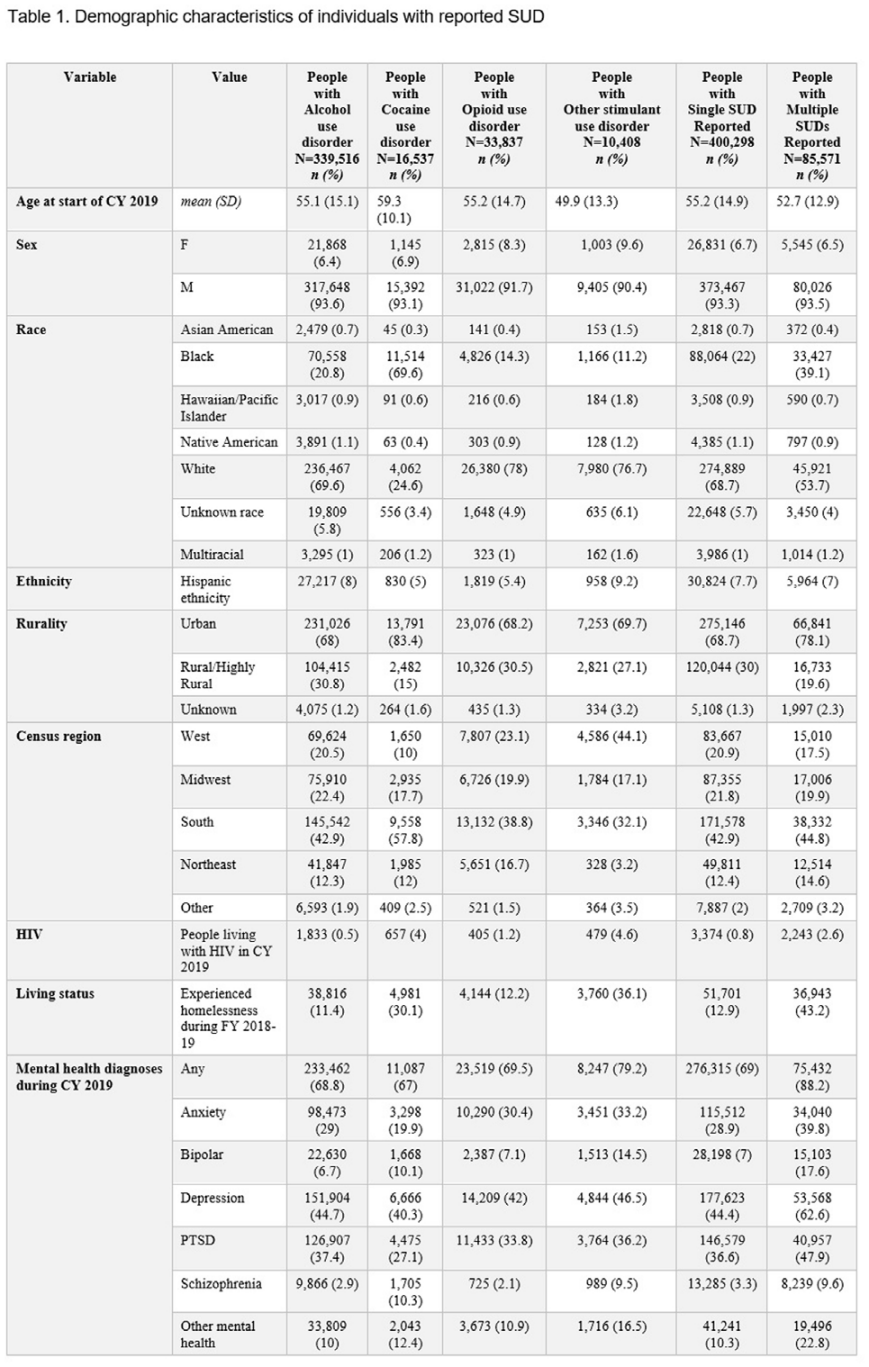

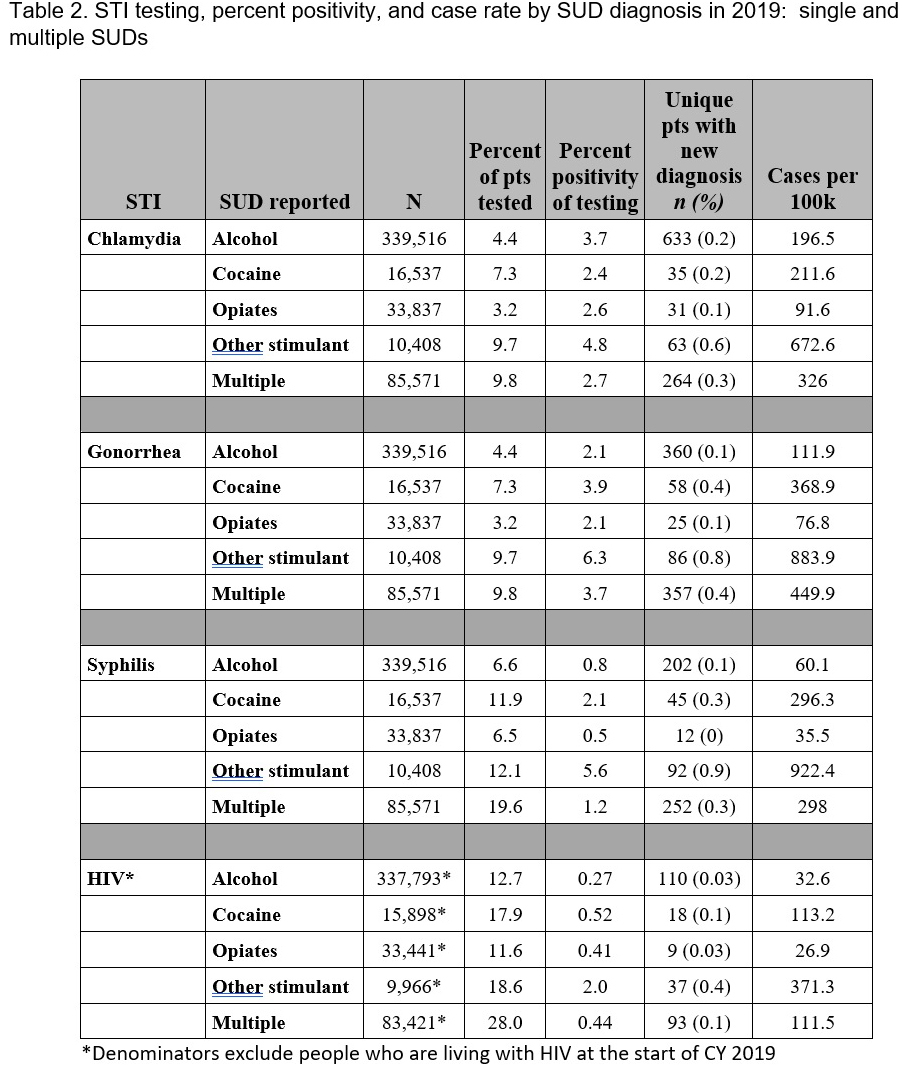

**Conclusion:**

High incidence of STIs among people with non-cocaine stimulant use disorder indicates a need for comprehensive testing. The data suggests that veterans with AUD would benefit from increased testing. Houselessness and mental health diagnoses were common, and comprehensive STI testing and treatment programs, including an assessment of HIV risk, should be integrated into programs addressing these comorbidities.

**Disclosures:**

**Holly Villamagna, MD**, Nothing to disclose

